# Supplementary material for: Pain and Cortisol in Patients with Fibromyalgia: Systematic Review and Meta-Analysis
Source: Diagnostics (Basel). 2020 Nov 9;10(11):922. doi: 10.3390/diagnostics10110922 (PMC7698032; doi:10.3390/diagnostics10110922)
Supplement: Supplementary file 1 [file diagnostics-10-00922-s001.pdf]

PRISMA 2009 Checklist

Section/topic	#	Checklist item	Reported on page #
**TITLE**			
Title	1	Pain and cortisol in patients with fibromyalgia: systematic review and meta-analysis	1
**ABSTRACT**			
Structured summary	2	Background<br>Some studies have shown a relationship between cortisol concentrations and levels of pain in centralized pain states such as fibromyalgia (FM). This systematic review and meta-analysis sought to gain further insight into the relationship between cortisol reactivity and chronic widespread pain in patients with FM.<br>Methods<br>To identify studies examining pain and cortisol responses to a therapeutic intervention, an electronic search was conducted of the databases Scopus, PubMed, ScienceDirect, PEDro, and WOS, using the search terms: (cortisol OR "cortisol level") AND (fibromyalgia OR "chronic fatigue syndrome" OR "chronic fatigue fibromyalgia syndrome") AND pain. The methodological quality of the studies was assessed using the PEDro scale. In a meta-analysis, we calculated effect sizes of interventions on pain indicators and cortisol levels in patients with FM.<br>Results<br>Of 263 studies identified, 12 were selected for our review and 10 were finally included as their methodological quality was good. A small overall effect of all the interventions was observed on pain tolerance and pressure pain thresholds yet this effect lacked significance (ES = 0.150; 95%CI 0.932-1.550; p > 0.05).<br>Conclusions<br>While some effects of individual nonpharmacological therapeutic interventions were observed on both cortisol levels and measures of pain, our results suggest much further work is needed to elucidate the true relationship between chronic widespread pain and cortisol levels in patients with FM.	1
**INTRODUCTION**			
Rationale	3	Identified the relation between cortisol and pain in patients with fibromyalgia is necessary for an adequate approach.	2
Objectives	4	Main goals were: a) to identify studies conducted in the past 10 years analyzing variations in cortisol levels and perceived pain produced in response to a treatment intervention or between subjects with and without FM; b) to assess the methodological quality of the studies identified; c) to calculate the effect sizes of the interventions proposed on cortisol and pain; and d) compare the effectiveness of the interventions.	2
**METHODS**			
Protocol and registration	5		
Eligibility criteria	6	Articles published in the last 10 years in English or Spanish; including the key words and dealing with the main topics investigated; including valid measurement tools.	2
Information sources	7	Databases: Pubmed, Science Direct, Scopus, Web of Science. Last searched December 2019.	2

PRISMA 2009 Checklist

Search	8	The search terms were obtained from MeSH through PubMed and included: cortisol, fibromyalgia and chronic fatigue, fibromyalgia syndrome and pain as follows: (cortisol OR "cortisol level") AND (fibromyalgia OR "chronic fatigue syndrome" OR "chronic fatigue fibromyalgia syndrome") AND pain.	2
Study selection	9	Of the 263 records initially identified in our review of the past 10 years, records excluded were 115 duplicates and 30 letters to the editor, notes, abstracts or meeting presentations. A further 7 articles were excluded because they were not written in English or Spanish. Another 79 articles were excluded because the main issue addressed was not related to fibromyalgia, cortisol and pain, and 22 because pain and cortisol variables were not provided. Finally, 12 articles fulfilling the inclusion and exclusion criteria were selected	2-3
Data collection process	10	Our review was conducted according to international PRISMA guidelines for systematic reviews and meta-analyses	3
Data items	11		
Risk of bias in individual studies	12	The positive bias caused by the standard deviation for small samples was treated through the corrected d value	3
Summary measures	13	Effect sizes of interventions were calculated on the outcome measures cortisol and pain. The data compiled were the means, standard deviations (SD) and the contrast statistics provided in the original articles (Student t, Mann Witney U and Snedecor F). Forest plots were constructed. A fixed effect model was employed to determine the homogeneity of the treatment measures of the original studies.	3
Synthesis of results	14	Of 263 studies identified, 12 were selected for our review and 10 were finally included as their methodological quality was good. A small overall effect of all the interventions was observed on pain tolerance and pressure pain thresholds yet this effect lacked significance (ES = 0.150; 95%CI 0.932-1.550; $p > 0.05$).	3

Section/topic	#	Checklist item	Reported on page #
Risk of bias across studies	15		
Additional analyses	16	Each of the 12 studies identified were scored according to the criteria of the PEDro scale	4
**RESULTS**			
Study selection	17	12 remained after applying the inclusion and exclusion criteria and eliminating repeated references in the different databases.	4

<table>
<tr><td></td><td></td><td>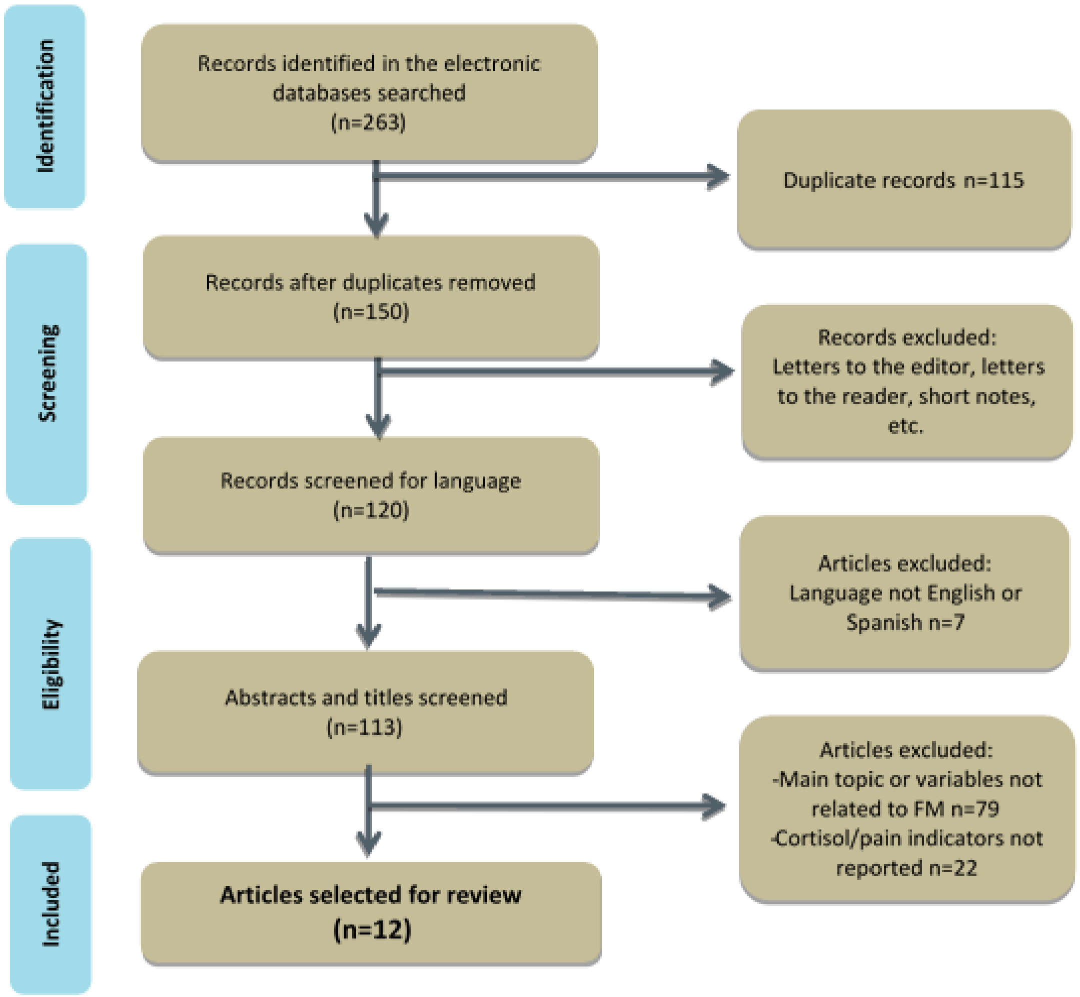
</td><td></td></tr>
<tr><td>Study characteristics</td><td>18</td><td>Data were extracted from all the articles that talked about the differences between men and women with fibromyalgia in all aspects.</td><td></td></tr>
<tr><td>Risk of bias within studies</td><td>19</td><td>For valid estimates of effect size for the meta-analysis model, the predictive variables compared were cortisol, pain tolerance threshold (PTT) and pressure pain threshold (PPT).</td><td></td></tr>
<tr><td>Results of individual studies</td><td>20</td><td></td><td></td></tr>
<tr><td>Synthesis of results</td><td>21</td><td>The methodological quality of the studies reported in these articles was good (PEDro score 5.60±1.07)</td><td>5</td></tr>
<tr><td>Risk of bias across studies</td><td>22</td><td></td><td></td></tr>
</table>

Additional analysis	23		
**DISCUSSION**			
Summary of evidence	24	This review and meta-analysis sought to summarize the latest scientific literature regarding the possible relationship between cortisol levels and the pain symptoms of fibromyalgia. The results of our meta-analysis indicate some individual effects of therapeutic interventions on both cortisol levels and several measures of pain though overall effect sizes were insignificant.	6
Limitations	25	There was also some heterogeneity detected, which indicated variation in the degree of association between the intervention tested and effects on pain and cortisol. The low number of studies means we were unable to provide a good estimate of the overall effect on cortisol and pain.	7-8
Conclusions	26	- The low number of clinical studies identified precludes establishing any clear relationship between cortisol levels and perceived pain when examining the effectiveness of several therapeutic interventions.<br>- The studies reviewed were of medium methodological quality thus revealing a need for higher quality trials conducted on larger patient numbers designed to examine variations in cortisol levels in response to pain in patients with fibromyalgia.<br>- The large number of different study designs identified in the literature led to similarly different effects observed. This variation, along with the different treatment interventions, could explain while overall effect sizes in relation to correlation between pain and cortisol did not prove significant.<br>- The scarcity of randomized controlled trials on this topic determines a need for much further work to elucidate the true relationship between chronic widespread pain and cortisol levels in patients with FM.	8-9
**FUNDING**			
Funding	27	We wish to confirm that there are no known conflicts of interest associated with this publication and there has been no significant financial support for this work that could have influenced its outcome.	8

From: Moher D, Liberati A, Tetzlaff J, Altman DG, The PRISMA Group (2009). Preferred Reporting Items for Systematic Reviews and Meta-Analyses: The PRISMA Statement. PLoS Med 6(7): e1000097. doi:10.1371/journal.pmed1000097

For more information, visit: **www.prisma-statement.org**.
